# Commentary: Anti-endothelial cell antibodies in pathogenesis of vasculitis

**DOI:** 10.3389/fimmu.2026.1750524

**Published:** 2026-03-09

**Authors:** Jiding Xie, Lei Shi, Leiyong Wang, Jingang Dai

**Affiliations:** 1 Experimental Research Center, China Academy of Chinese Medical Sciences, Beijing, China; 2 College of Physical Education, Yangzhou University, Yangzhou, Jiangsu, China; 3 Beijing Hospital of Traditional Chinese Medicine, Capital Medical University, Beijing, China

**Keywords:** AECAs, chapel hill consensus, endothelial injury, endotype, mechanism-informed classification, nomenclature, vasculitis

## Abstract

Recent analyses of anti-endothelial cell antibodies (AECAs) have renewed interest in the immunological pathways underlying vascular inflammation. Although endothelial injury mediated by AECAs constitutes a clearly defined mechanistic contributor to a subset of vasculitic syndromes, accumulating evidence from virology, autoinflammation, complement biology, molecular genetics, and tissue injury suggests that vasculitis is better conceptualized as a final common pathological endpoint rather than a uniformly immune-mediated disease. In this perspective, we argue that the traditional immune-centric definition of vasculitis fails to encompass this mechanistic diversity. We outline multiple upstream mechanisms — including infection-driven, immune-complex–mediated, autoinflammatory, complement-mediated, monogenic, and injury-induced pathways — and propose a mechanism-informed nomenclature that integrates conventional vessel-size–based classification with pathway-based endotypes. This dual-layer approach may enhance diagnostic precision, improve biomarker interpretation, guide targeted therapeutic strategies, and better align vasculitis terminology with contemporary biological understanding.

## 1 Introduction

The recent review by Zhang et al. on anti-endothelial cell antibodies (AECAs) provides a timely synthesis of how endothelial-directed autoantibodies contribute to vascular inflammation (1). By integrating evidence on endothelial activation, inflammatory amplification, and vascular injury, their work highlights a coherent immunological pathway that characterizes a subset of vasculitic syndromes.

This mechanistic clarity is valuable, as it illuminates one of the routes through which vascular inflammation can arise. At the same time, the AECA-centered perspective presented in the review raises a broader conceptual question: does defining vasculitis primarily as an immune-mediated disorder fully capture the biological heterogeneity now recognized across these conditions?

The mechanistic patterns illustrated by Zhang et al. underscore an important point — namely, that autoantibody-mediated endothelial injury represents only one among several distinct pathways capable of producing a vasculitic phenotype. As our understanding of infection-driven, autoinflammatory, complement-mediated, monogenic, and injury-related mechanisms expands, the traditional immune-centric definition of vasculitis appears increasingly insufficient.

Rather than contradicting the authors’ conclusions, this perspective extends their insights: it suggests that current vasculitis nomenclature may benefit from incorporating mechanistic specificity alongside phenotypic classification. Doing so may better align terminology with the true biological diversity of vasculitic syndromes and improve diagnostic precision and therapeutic decision-making.

This editorial is intended as a conceptual extension to the valuable framework outlined in the authors’ review and aims to situate AECA-related mechanisms within the broader landscape of vasculitis biology. We also discuss practical implementation constraints and a transitional strategy to avoid over-inference from endotyping and single biomarkers at the current stage.

## 2 Vasculitis as a final common pathway rather than a unified etiologic entity

For much of the 20th century, vasculitic syndromes were identified and named through the lens of eponyms, honoring the physicians who first described distinctive clinical clusters, such as Wegener’s granulomatosis and Churg-Strauss syndrome.

These eponymous designations served an important historical purpose, providing shorthand labels for complex multisystem presentations lacking known causes. However, such nomenclature offers no insight into underlying pathophysiology.

The Chapel Hill Consensus Conference (CHCC) framework has long organized vasculitides by vessel size and histopathology, providing an indispensable structure for diagnosis and research (2). Yet advances in immunology, virology, genetics, and multi-omic profiling increasingly reveal that vasculitis is less an etiologic disease category than a final common pathological endpoint—a vascular inflammatory phenotype produced by multiple distinct upstream mechanisms.

This reconceptualization has practical consequences, influencing biomarker interpretation, therapeutic strategy, prognosis, and causal reasoning. While AECA-mediated endothelial injury represents one clearly delineated pathway, many others converge toward similar vascular inflammation.

Other mechanistic pathways include:

- Persistent viral infection, such as hepatitis C–associated cryoglobulinemic vasculitis (3)
- Immune-complex–mediated vascular inflammation (4)
- Autoinflammatory activation (e.g., IL-1β/IL-6–associated neutrophilic hyperreactivity in Behçet’s syndrome) (5)
- Complement dysregulation (6)
- Direct structural injury (e.g., radiation-induced vasculopathy) (7)
- Monogenic enzymatic defects (e.g., DADA2) (8)

These diverse origins converge clinically toward vascular inflammation, illustrating vasculitis as a shared endpoint rather than a unified etiologic category.

## 3 AECA-associated conditions exemplify one mechanistic subset

The diseases discussed in the review—including Takayasu arteritis, giant cell arteritis, Kawasaki disease, IgA vasculitis, Behçet’s syndrome, ANCA-associated vasculitis, SLE-related microvascular inflammation, and sarcoidosis—share a common feature: vascular inflammation driven, at least in part, by immune-mediated endothelial injury.

In this subset, AECAs may act as direct effectors or facilitators of endothelial dysfunction, promoting leukocyte recruitment, vascular permeability, and inflammatory signaling.

These entities may therefore be conceptualized as:

Autoantibody-associated, immune-mediated endothelial vasculopathies presenting with vasculitic syndromes.

## 4 Diverse mechanistic routes challenge an exclusively immune-mediated definition

### 4.1 Infection-driven vasculitis

Persistent viral replication (e.g., HCV) drives immune-complex deposition and vascular injury. Vasculitis often resolves with antiviral therapy, indicating immune activation is secondary.

### 4.2 Drug-induced vasculitis

Drug-induced small-vessel vasculitis and ANCA vasculitis represent reversible disruptions of immune tolerance rather than primary autoimmune diseases.

### 4.3 Autoinflammatory vasculitis

Neutrophilic hyperreactivity and IL-1β/IL-6 dysregulation in conditions like Behçet’s syndrome align more closely with autoinflammatory disorders.

### 4.4 Non-immune instigators

Radiation injury and monogenic defects such as DADA2 demonstrate vasculitic pathology independent of adaptive immunity.

Together, these examples illustrate that immune mediation is only one of several foundational mechanisms.

## 5 AECA-related pathways underscore the need for mechanistic taxonomy

AECAs are detectable in a subset of vasculitic diseases and are mechanistically meaningful only in specific contexts. These observations support a classification system integrating pathway-based endotypes alongside phenotypic categories.

## 6 A proposed mechanism-informed nomenclature

Layer 1 — Phenotypic category

- Large-vessel vasculitis
- Medium-vessel vasculitis
- Small-vessel vasculitis
- Variable-vessel vasculitis

Layer 2 — Mechanistic endotype

Examples include:

- Autoantibody-mediated endothelial endotype (AECA-associated)
- ANCA-mediated neutrophil-activation endotype
- Autoinflammatory IL-1β/IL-6 endotype
- Infection-driven immune-complex endotype
- Monogenic ADA2-deficiency endotype
- Radiation-associated endothelial-injury endotype

Combined examples

- Large-vessel vasculitis, AECA-associated endothelial autoantibody endotype
- Small-vessel vasculitis, ANCA-associated neutrophil-activation endotype
- Medium-vessel vasculitis, IL-1β-driven autoinflammatory endotype

Importantly, these endotypes are not intended as mutually exclusive bins. In practice, mechanisms often overlap and evolve over time. We therefore describe endotyping as identifying a dominant driver mechanism with potential secondary modifiers, explicitly allowing mixed/composite endotypes when supported by convergent evidence (e.g., infection-triggered autoimmunity including AECA generation with downstream complement amplification).

This layered structure:

1. Differentiates pathological endpoints from initiating biology
2. Reduces biomarker misinterpretation
3. Aligns nomenclature with precision therapeutics
4. Anticipates multi-omic stratification

## 7 Clinical implications of mechanism-informed naming

### 7.1 Therapeutic precision

Mechanistic labels guide therapy selection: antivirals for infection-driven vasculitis, IL-1 inhibition for autoinflammatory pathways, B-cell depletion for antibody-mediated disease.

### 7.2 Biomarker interpretability

AECA positivity is most interpretable only in disease contexts where endothelial autoimmunity plausibly represents a dominant driver. However, current AECA assays remain insufficiently standardized and lack harmonized reference standards, diagnostic performance varies across platforms/cohorts, and titers do not consistently correlate with clinical activity. Moreover, non–endothelial-restricted antigen expression increases cross-reactivity; thus, AECAs should be used as an adjunctive signal integrated with other immunologic and functional evidence.

### 7.3 Research stratification

Endotypes facilitate pathway-specific trials and biomarker discovery.

### 7.4 Communication clarity

Mechanistic terminology improves conceptual alignment between clinicians and investigators.

### 7.5 Implementation considerations and current limitations

A key practical barrier is that mechanistic endotypes cannot always be assigned at first presentation because endotype-defining biomarkers are not yet universally available, cost-effective, or standardized. To improve feasibility, we propose an evidence-tiered, stepwise workflow: a suspected endotype based on phenotype and routine laboratory/imaging patterns, refinement to a probable endotype using targeted immunologic assays and pathology where appropriate, and a confirmed endotype supported—when feasible—by functional readouts and/or multi-omic evidence. During this transition, an “endotype-undetermined/unknown” label may persist, but it remains useful by guiding structured work-up, preventing overinterpretation of single biomarkers, and enabling consistent stratification for research and trials.

We further clarify boundaries for AECA interpretation: current assays are insufficiently standardized (antigen sources, platforms, thresholds, and reporting), diagnostic performance varies across cohorts, and titers do not consistently correlate with disease activity. In addition, many reported AECA targets are not endothelial-restricted and may be expressed on fibroblasts or leukocyte-lineage cells, increasing cross-reactivity and reducing specificity. Accordingly, AECAs should be interpreted as a context-dependent adjunct and/or mechanistic research tool, integrated with phenotype, complementary immunologic evidence, and—when feasible—cell-based functional endothelial readouts, rather than serving as a standalone diagnostic or monitoring metric.

## 8 Proposed refinement of terminology for AECA-related diseases

To reflect mechanistic coherence, we suggest the term:

“AECA-associated immune-mediated endothelial vasculopathies presenting with vasculitic syndromes.”

This nomenclature reflects the biological patterns while avoiding overextension of AECA relevance.

## 9 Conclusion

Zhang et al.’s review is a timely synthesis of the role of AECAs in immune-mediated endothelial injury. At the same time, it illustrates that vasculitic syndromes arise from multiple mechanistic pathways.

Integrating these pathways into formal nomenclature may enhance diagnostic accuracy, therapeutic precision, and conceptual coherence. The mechanism-informed terminology proposed here aims to complement ongoing developments in vasculitis classification.Future work should focus on assay harmonization and prospective validation of endotype-guided workflows.
